# Dual THz Wave and X-ray Generation from a Water Film under Femtosecond Laser Excitation

**DOI:** 10.3390/nano8070523

**Published:** 2018-07-13

**Authors:** Hsin-hui Huang, Takeshi Nagashima, Wei-hung Hsu, Saulius Juodkazis, Koji Hatanaka

**Affiliations:** 1 Research Center for Applied Sciences, Academia Sinica, Taipei 115, Taiwan; hsinhuih@gate.sinica.edu.tw (H.-h.H.); jacky81418@gate.sinica.edu.tw (W.-h.-H.); 2 Faculty of Science and Engineering, Setsunan University, 17-8 Ikeda-Nakamachi, Neyagawa, Osaka 572-8508, Japan; 3 Nanotechnology Facility, Center for Micro-Photonics, Swinburne University of Technology, Hawthorn, VIC 3122, Australia; 4 Melbourne Centre for Nanofabrication, the Victorian Node of the Australian National Fabrication Facility, Clayton, VIC 3168, Australia; 5 College of Engineering, Chang Gung University, Taoyuan 33302, Taiwan; 6 Department of Materials Science and Engineering, National Dong-Hwa University, Hualien 97401, Taiwan

**Keywords:** femtosecond laser, intense laser, water, THz wave, time-domain spectroscopy, X-ray, ablation, double-pulse excitation, plasma, z-scan, intensity enhancement

## Abstract

Simultaneous emission of the THz wave and hard X-ray from thin water free-flow was induced by the irradiation of tightly-focused femtosecond laser pulses (35 fs, 800 nm, 500 Hz) in air. Intensity measurements of the THz wave and X-ray were carried out at the same time with time-domain spectroscopy (TDS) based on electro-optic sampling with a ZnTe(110) crystal and a Geiger counter, respectively. Intensity profiles of the THz wave and X-ray emission as a function of the solution flow position along the incident laser axis at the laser focus show that the profile width of the THz wave is broader than that of the X-ray. Furthermore, the profiles of the THz wave measured in reflection and transmission directions show different features and indicate that THz wave emission is, under single-pulse excitation, induced mainly in laser-induced plasma on the water flow surface. Under double-pulse excitation with a time separation of 4.6 ns, 5–10 times enhancements of THz wave emission were observed. Such dual light sources can be used to characterise materials, as well as to reveal the sequence of material modifications under intense laser pulses.

## 1. Introduction

An intense (>10${}^{13}$ W/cm${}^{2}$) femtosecond laser at near-IR (photon energy ∼1 eV) and matter interaction [1,2,3] induce highly-nonlinear optical processes and result in photon conversion to an X-ray of several-keV [4], as well as to a THz wave at meV [5] in association with the white light continuum in the visible spectral range [6]. Strong laser ablation of solid and solution targets also occurs, which is usually associated with the production of laser plasma [7]. Studies of such photon conversion mechanisms for different applications [8,9,10] have been carried out separately for widely different wavelengths. Experimental techniques based on the X-ray or THz wave have been contributing immensely to basic physics and chemistry/material science [10,11,12,13], have expanded the understanding of the basic mechanisms of intense laser-matter interaction and have contributed to laser-material processing/printing [14].

Gases [15,16,17,18], atomic clusters [19,20,21] and solids [22,23] have been used as targets for THz wave emission from the laser-induced plasmas. Fast electron motions accelerated by laser ponderomotive forces [15,18,19] and the four-mixing process induced by two-colour excitation [17] have been proposed as THz emission mechanisms from laser-induced plasmas. Although such laser-induced plasmas of the solid targets generate a powerful THz wave because of the high plasma densities, there are problems with the instability of the intensity and a limitation in the successive delivery of laser irradiation to the target. The use of liquids or solutions as targets is promising since solutions have moderate atomic density (∼$10^{22}$ cm${}^{-3}$) and ease of the delivery, but there have been no reports with liquid targets (water) until recently [5,24]. As for intense laser-induced X-ray emission, it has been widely studied with solids [15,25,26,27,28] and solutions [29,30,31,32,33,34,35,36,37,38]. One advantage of using solutions, e.g., water, as a target sample is that a fresh and smooth surface can be easily prepared for each laser irradiation even under intense laser irradiation at high repetition rates. Furthermore, the addition of solute chemical agents such as electrolyte, CsCl for instance [39], with different concentrations or nano-particles, gold for instance [40], with different shapes and sizes into water makes it possible to explore different excitation/absorption mechanisms and to control the spectral characteristics of X-ray sources by utilizing solute-dependent monochromatic characteristic X-ray lines and broad components mainly based on bremsstrahlung.

Simultaneous emission of the THz wave and X-ray and combined usages of THz wave and X-ray pulses have recently been introduced as readily-available table-top realisations [41]. To date, experiments on such simultaneous emission with a Al-coated glass substrate [15] and gas targets, He [15] or Ar [20], in vacuum chambers were reported. This type of work, though the numbers of such reports are still quite limited, will be the first step for the discussion of the conversion of near-IR (eV) laser photon energy into photons at the two ends of the spectrum, several-keV (X-ray) and meV (THz wave), and will contribute well to other fields like material sciences. One important parameter for the synchronized emission of the X-ray and THz wave for practical applications is their intensities. It has been reported that chirped femtosecond laser pulses enhance THz wave emission from water [24] and the X-ray from aqueous solutions [42]. In cases of X-ray emission from aqueous solutions, double-pulse excitations [37,43] and the addition of solutes such as electrolytes [37] or gold nano-particles [40] to water are also reported to be effective for the X-ray intensity enhancements. For THz wave emission, one recent experimental study with double-pulse excitation to gas-clusters in a vacuum chamber [21] was reported, but the inter-pulse delay time was limited to 0.5 ns. One advantage of the solution targets, in addition to that described above, is the possibility of a transient solution surface roughening from its original nano-smooth surface by a pre-pulse irradiation. Dynamic changes induced on the liquid surface by plasma formation, capillary transient surface roughening instabilities and mist/droplet formation associated with shock-wave expansion [44] contribute favourably to increased interaction volume and augmented X-ray intensity up to more than an order of magnitude [37,43].

In this study, a simultaneous emission of the THz wave and hard X-ray in air using distilled water as a target irradiated by tightly-focused near-IR femtosecond laser pulses and THz wave emission enhancements under double-pulse excitation are presented.

## 2. Experimental Section

**Figure 1:**
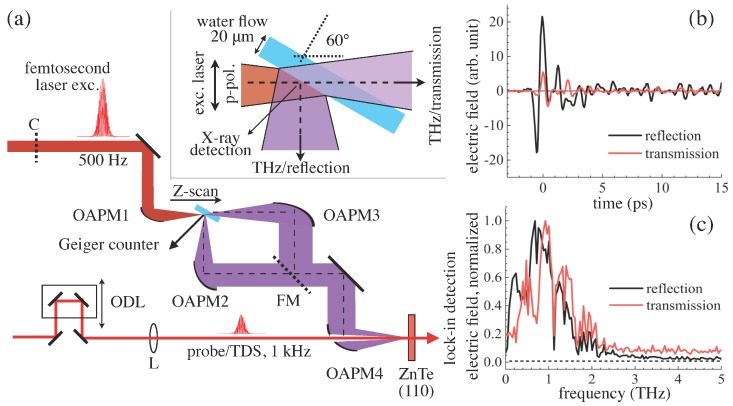
(**a**) The experimental setup for the simultaneous measurements of THz wave (time-domain spectroscopy (TDS)) and X-ray (Geiger counter). The femtosecond laser pulses (<35 fs, 800 nm, 500 Hz, horizontally-polarised to the solution flow surface, i.e., p-pol.) were focused onto the solution flow; ODL, optical delay line for TDS, L-plano-convex lens (f=50 cm). The thickness of ZnTe(110) crystal for TDS was 1 mm; C, optical chopper (500 Hz) for TDS measurements. The parent focal lengths for off-axis parabolic mirrors (OAPMs) (off-axis parabolic mirrors) were *f* = 50.8 mm (OAPM1, 1-inch diameter), 101.6 mm (OAPM2, 2-inch), 152.4 mm (OAPM3, 2-inch) and 101.6 mm (OAPM4, 2-inch with a hole in its centre for the probe), respectively. The distance between the laser focus and Geiger counter was 12 cm. FM, flip-folding mirror for TDS measurements in the transmission direction. The inset shows the close-up of the solution surface, the laser-water interaction region; (**b**) Representative TDS signals from water flow when the laser intensity is 0.4 mJ/pulse in reflection and transmission directions at the Z-position for the highest X-ray intensity and (**c**) their normalized spectra.

The experimental setup is shown in Figure 1a. A flat solution flow of distilled water with a thickness smaller than 20 μm was prepared using a metal nozzle (Flatjet Nozzle LARGE, Metaheuristic, Okayama, Japan), and the flow rate was regulated by a circulation pump (PMD-211, SANSO, Hyogo, Japan) controlled by a conventional voltage regulator. The flow rate was set at <70 mL/min. The nozzle was mounted to a rotational and 3D-automatic stages (KS701-20LMS, Suruga Seiki, Shizuoka, Japan), and its position was finely set by a home-made LabView code, as reported previously [43]. Transform-limited femtosecond laser pulses (λ = 800 nm, >35 fs, 1 kHz, linearly-polarized, Mantis, Legend Elite HE USP, Coherent, Inc., Santa Clara, CA, USA) were separated as two beams with different polarizations by a half-wave plate (65-906, Edmund Optics, Barrington, NJ, USA) and a polarization beam splitter (47-048, Edmund Optics, Barrington, NJ, USA). Horizontally- and vertically-polarized pulses were defined as the excitation pulse for X-ray/THz wave generation and the probe pulse for the time-domain spectroscopy (TDS) for THz wave measurements [45,46], respectively. The repetition rate of the excitation pulses was modulated by a wheel chopper (500 Hz, 3502 Optical Chopper, New Focus, CA, USA) for TDS measurements, and the pulses were tightly-focused in air onto the solution flow surface by using an off-axis parabolic mirror (OAPM, 1-inch diameter, the effective focus length f=50.8 mm, the reflection angle of 90 degrees and numerical aperture NA=0.25, 47-097, Edmund Optics, Santa Clara, CA, USA). The incident angle of the excitation pulses along the *Z* -axis to the solution normal was fixed at 60 degrees for the highest X-ray emission, as reported previously [42]. Under these experimental conditions, each excitation pulse at a 500-Hz repetition rate irradiates the fresh and flat solution flow. Experiments on double-pulse excitations with a pre-pulse (vertically-polarized, 0.1 mJ/pulse, 4.6 ns in advance of the main excitation pulse) were also carried out using an optical delay line (SGSP46-800, Sigma Koki, Tokyo, Japan).

X-ray intensity was measured by a Geiger counter (SS315, Radhound, southern scientific, West Sussex, UK). All the measurements were carried out in air under atmospheric pressure (1 atm) at room temperature (RT = 296 K). Its observation angle was 15 degrees to the solution normal towards the excitation side, and its distance from the laser focus was 12 cm. Therefore, it is certain that the Geiger counter detects only X-ray, neither α- nor β-ray. THz wave signals were collected in reflection (30 degrees to the solution normal, 90 degrees to the laser incident direction) and transmission (along the excitation *Z*-axis) directions with two independent OAPMs (the reflected focal length f=101.6 mm and 152.4 mm, the off-axis angle of 90 degrees, MPD249-M01, MPD269-M01, ThorLabs, Newton, NJ, USA). As conventional TDS measurements, THz wave and the probe pulses after n variable optical delay (TSDM60-20, OptoSigma, Tokyo, Japan) were focused to a 1 mm-thick ZnTe(110) crystal (Nippon mining & metals Co., Ltd., Tokyo, Japan) by an OAPM (the reflected focal length f=101.6 mm, the off-axis angle of 90 degrees, MPD249H-M01, ThorLabs, Newton, NJ, USA) and a plano-convex lens (f=50 cm), respectively. Lock-in measurements were carried out with a balanced photo-diode (Model2307, New Focus, CA, USA) and a lock-in amplifier (SR830, Stanford Research System, Sunnyvale, CA, USA). For measurements in the transmission direction, an additional flip-folding mirror (FM) was set for the THz wave path to be bent; therefore, the total number of metal mirrors for the transmission measurements was larger by one compared to that for the reflection.

One representative THz wave signal of water in reflection/transmission directions by the EO sampling in TDS measurements is shown in Figure 1b. Detection efficiencies for the reflection and the transmission directions, though their optical paths were shared partly, have not been calibrated; therefore, the THz wave intensities and converted FFT spectra are not comparable quantitatively between the signals in the two observation directions. A single cycle of the electric field oscillation was clearly observed, and the vibration structure afterwards was also very distinct. This is reflected in their Fourier-transformed emission spectra shown in Figure 1c as absorption bands at 1.1 and 1.4 THz due to water vapour, as reported elsewhere [47]. Note that the spectra shown in Figure 1c are normalized. This absorption is considered to be due to the water vapour in the atmosphere of the laboratory and long-living micro-droplets (mist) formation in the vicinity of the water film induced by the laser irradiation every 2 ms. The central frequencies of the observed THz wave were around 0.9–1 THz for the reflection and the transmission. As discussed in detail below, the THz wave was emitted from the area in the vicinity of the upstream-side of the air/water interface. In the transmission, the THz wave transmitted through the water film. Water absorption in the THz wave region was well studied recently, and a transmission experiment of the THz wave through 0.5 mm-thick water films was recently demonstrated using TDS for precise measurements of temperature [48], the discussion of which can be used for characterization of light-water interaction at high intensities. Under conventional transmission conditions thorough 20 μm-thick water toward the transmission direction, but with the incident angle at 60 degrees, the transmittance of the THz wave electric field intensity at 1 THz can be estimated to be 0.64. Since water shows higher absorption at higher frequencies [48,49], the high frequency components in the transmission are expected to be reduced compared with those in the reflection. However, the observed spectra in the transmission showed a slight blue shift compared to that in the reflection. It should be pointed out that the small shift can be due to an extrinsic effect since the spectra obtained in the transmission and the reflection directions have not been calibrated in this study. In the following, the relative intensities of the THz wave obtained in the identical direction are discussed.

Under these conditions, X-ray and THz wave emission was induced at the same time and measured simultaneously. Experiments were performed with different excitation laser intensities, different solution positions along the *Z*-axis and under the double-pulse excitation. All the experiments were carried out at atmospheric pressure at RT conditions.

## 3. Results and Discussion

### 3.1. Laser Intensity Dependences

**Figure 2:**
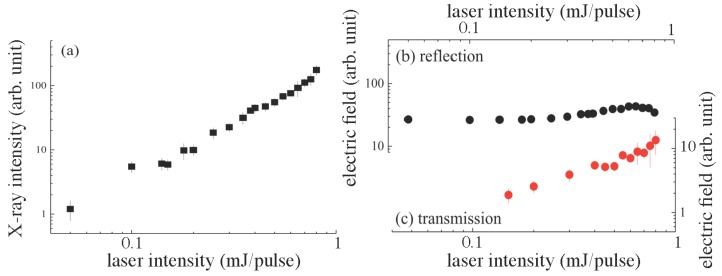
Laser intensity dependencies of (**a**) X-ray and (**b**) THz wave intensities. The laser polarization was horizontal to the solution flow, i.e., p-pol. During the measurements, the solution flow position along the *Z*-axis was finely optimized for the highest X-ray intensity at each laser intensity.

Figure 2 shows the intensities of the X-ray and THz wave in the reflection and the transmission as a function of the excitation laser intensity. The solution flow position along the *Z*-axis was optimized for the highest X-ray intensity for each laser intensity. The highest intensity of X-ray was obtained when the highest electron temperature, $T_{e}$, was reached, hence for the strongest absorption of femtosecond laser pulses. The X-ray emission in Figure 2a shows the slope γ=2 scaling for this maximized absorbed intensity. The absorbed energy density, $W_{abs}$, (per volume) is the relevant quantity that should be considered, since the free electron density, $n_{e}$, is approaching the critical density, $n_{cr}$, (plasma reflection range) during the light absorption and $W_{abs}\propto \frac{n_{e}}{n_{cr}}F_{p}$, where $F_{p}$ is the pulse fluence [43]. The absorbed energy density was defined by the mechanism of electron generation $n_{e}\propto I_{p}^{m}\propto F_{p}^{m}$ and determined by the corresponding exponent *m* (m=1 for the linear absorption). The strongest absorption took place when permittivity ϵ⇒0 when material was transforming from dielectric to metal-like [50]. Under these conditions, direct light absorption m=1 was dominant regardless of the initial nonlinearity required to excite free carriers and to decrease the real part of the permittivity. The experimentally observed slope γ=2 shown in Figure 2a of the X-ray intensity as a function of the excitation laser intensity energy $E_{p}$ (or $I_{p},F_{p}$) was expected. This signifies a decreasing volume where light was absorbed at the increasing electronic excitation. This was confirmed by femtosecond laser ablation and etching where strong localization of modification took place at the very centre of the laser irradiated spot [51].

THz wave intensities measured in the reflection and the transmission are shown in Figure 2b,c, respectively. For the 20 μm-thick solution flow, it showed different behaviours: THz wave emission in the reflection was almost independent of the pulse intensity, while that in transmission was linearly increasing $E_{THz}\propto E_{p}$ for the p-polarized laser excitation. At the used NA=0.25, the geometric focus diameter was d=1.22λ/NA∼3.9μm, which for the smallest excitation laser intensity $E_{p}=0.1$ mJ defined the intensity $I_{p}=E_{p}/\left(\pi {\left(d/2\right)}^{2}t_{p}\right)=24$ PW/cm${}^{2}$. The actual pulse intensity was considerably lower than this calculation due to air breakdown and intensity clamping known in the filamentation of femtosecond pulses [43]. Such high irradiance conditions are usually not explored in the studies of THz wave emission, and low irradiance scaling is usually $P_{THz}\propto P_{l}^{2}$ [52].

There have been controversial and various discussions on THz wave emission mechanisms from laser-induced plasmas. As the first step for such discussions on water, however, it is meaningful to learn discussions on similar experimental conditions with metal targets [52] to those in this paper with water; under a grazing angle laser excitation, with flat solution flow and the THz wave radiation from the flow surface. THz wave emission from solid flat surfaces under a grazing angle excitation, α, (angle between the surface and the laser beam) was usually found to be much longer at ∼1 ps (1 THz) than the excitation ultra-short laser pulse of tens of femtoseconds; the angle of incidence was (α∼π/2) [53]. The excitation laser pulse was travelling on the surfaces at the velocity v=c/cosα, and the heated electrons emitted the THz field $E_{THz}\propto \partial T_{e}/\partial t\propto E_{⊥}/\sin\alpha$, where $E_{⊥}$ is the light field component perpendicular to the solid surface (*p*-component of the laser E-field), $T_{e}$ is the electron temperature and *c* and *t* are the speed of light and time, respectively [52]. This Cherenkov-type synchronism [52] explains THz wave emission in specular reflection, its polarization (defined by $E_{⊥}$) and the angular dependence of the THz wave emission pattern on the light incidence angle for the *s* and p-polarisations. The electron scattering mechanisms and their temperature dependence define the strongly nonlinear scaling of the emitted THz power $P_{THz}\propto P_{l}^{4/(2-n)}$, where $P_{l}$ is the laser pulse power and *n* (n<2) is the exponent defining the temperature dependence of the effective electron collision frequency $\nu =\nu_{0}+\beta T^{n}$ [52]. Arguably, in the case of water, the plasma breakdown on its surface acts like a metal mirror for the THz wave generated in the air breakdown region, as well as from a plasma skin depth travelling on the flow surface at the slanted irradiation, as discussed above (see the inset in Figure 1a). The transmission of the THz wave was weaker, but was linearly increasing with the excitation laser intensity, as shown in Figure 2c. This is an indication of either a larger excited volume or a higher temperature of electrons (considering that there was no strong difference in electron scattering behaviour). The larger excited volume emitting the THz wave was one probable cause, since the dependence of the THz wave emission on the axial position of optical femtosecond laser excitation on the solution flow showed a wider axial width, as discussed next.

### 3.2. Z-Scan for THz Wave and X-ray Emission

**Figure 3:**
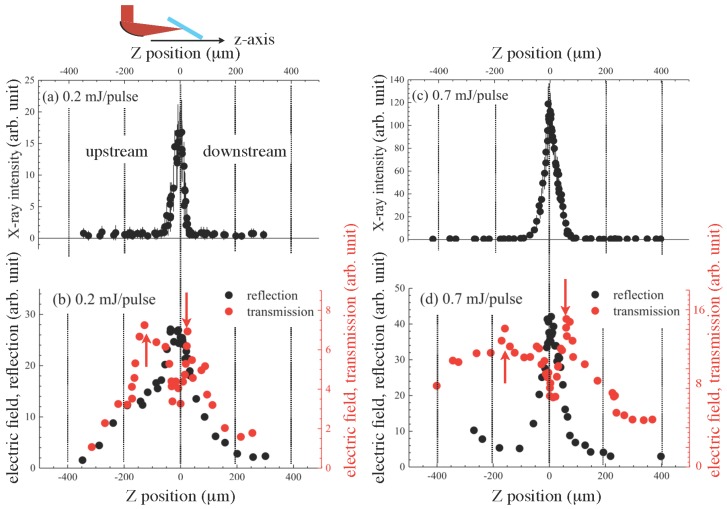
X-ray (**a**) and THz wave (**b**) intensities as a function of the flow position along the *Z*-axis when the laser intensity was 0.2 mJ/pulse and 0.7 mJ/pulse (**c**,**d**); Red arrows in (**b**,**d**) for the transmission indicate the Z-positions at (**b**) −135 μm and 24 μm ($\Delta z_{1}$ = 159 μm) and (**d**) −167 μm and 62 μm ($\Delta z_{2}$ = 229 μm), respectively.

Figure 3 shows the X-ray and THz wave intensity profiles at different solution positions along the *Z*-axis when the excitation laser intensities were 0.2 and 0.7 mJ/pulse. The X-ray profile (Figure 3a) showed an asymmetric feature with a longer tail to the upstream side. This reflects that the X-ray emission mechanism was mainly related to laser-induced plasma formation [25]. When the solution was set at the downstream side, the incident laser light was partly reflected by the self-induced plasma, which resulted in the instantaneous degradation of X-ray intensity observed in the downstream side. When the excitation laser intensity increased to 0.7 mJ/pulse (Figure 3c), the profile width became broader from 44 μm to 50 μm with a broader tail, and this may indicate that the X-ray source size became enlarged. X-ray emission spectra in the hard X-ray region from water under these experimental conditions [38,39] showed a broadband emission due to bremsstrahlung with no characteristic lines since such bands of oxygen and hydrogen are far in the longer wavelength region [54] and the estimated electron temperature, $T_{e}$, was up to 2 keV at the highest [38,39].

The peak position of THz wave emission in the reflection direction (Figure 3b) was almost the same as that of X-ray emission though its width of THz wave emission was apparently broader at 225 μm compared with X-ray emission. This implies that there is a mechanism to enhance the THz emission from the water plasma with low electron density and temperatures when the focal point is far from the water film. One possible mechanism is the four-wave mixing/optical rectification process [17] in which the second harmonic (400 nm) component of the white light continuum was generated in air at the focal point by self-phase modulation and the residual excitation laser pulse was mixed, which resulted in the enhancement of THz wave emission from the water plasma at the far sides from the peak position. On the other hand, the profile width when the laser intensity was 0.7 mJ/pulse (Figure 3d) became as narrow as the width of the X-ray emission, which indicates that the mechanism of laser-induced plasma formation dominated more in THz wave emission when the laser intensity increased. THz wave emission from only air was negligibly small; however, when the solution position was set far from the laser focus z>200μm at the downstream side, the emission became relatively dominant, as shown in Figure 3b.

In the transmission, THz wave emission changed its nature from that in the reflection. When the laser intensity was 0.2 mJ/pulse, intensity degradation at the peak position for THz wave emission in the reflection was clearly observed (Figure 3b). In addition, local peaks at the upstream side (z=−135μm) and the downstream side (z=24μm) were observed as indicated by red arrows. This feature may be assigned to the laser-induced plasma especially, at Z-positions close to the THz wave emission peak in reflection; the laser-induced plasma reflected THz wave radiation. Under this hypothesis, at the positions indicated by the red arrows, the plasma density reached its critical density for THz wave, defined as $\omega_{p}=\sqrt{n_{e}e^{2}/m_{e}\varepsilon_{0}}$, where $\omega_{p}$, $n_{e}$, *e*, $m_{e}$ and $\varepsilon_{0}$ are the plasma cyclic frequency, electron density, its mass and permittivity. The critical electron density $n_{e}=n_{cr}$ was $1.23\times 10^{16}$ cm${}^{-3}$ for 1 THz. The Debye length, $\lambda_{D}$, could be also estimated to be about 2.3 μm with $T_{e}$ at 2 keV [38,39]. Similarly, when the laser pulse energy increased to 0.7 mJ/pulse (Figure 3d), such features were clearly observed, and the position range along the Z-axis with the plasma density higher than critical $n_{cr}=1.23\times 10^{16}$ cm${}^{-3}$ was changed from $\Delta z_{1}=159\;\mu$m (0.2 mJ/pulse) to $\Delta z_{2}=229\;\mu$m (0.7 mJ/pulse) along the *Z*-axis. As in the discussion of Figure 3b described above, the broad tails at the upstream and the downstream sides can be assigned to optical coherent processes such as four-wave mixing. Its relative intensity at the tails to the intensity close to zero-position was increasing if compared to the case with 0.2 mJ/pulse (Figure 3b), which was considered to be the result of the dominant plasma effect at the zero-position when the laser intensity was higher. Furthermore, similarly to the case of the reflection shown in Figure 3b, THz wave emission from air became dominant when the solution position was far from the laser focus and was observed as the constant THz wave intensity at the solution position z>250μm at the downstream side. For all the excitation laser intensities, the high reflectivity correlated with low transmittance of the water film target.

### 3.3. THz Wave Intensity Enhancements under Double-Pulse Excitation

**Figure 4:**
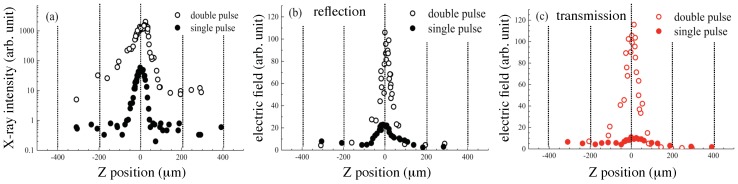
Z-position-dependent intensities of the X-ray in log-scale (**a**), the THz wave in the reflection (**b**) and the transmission (**c**) under the double-pulse excitation condition. Filled circles are under the single-pulse excitation condition, and open circles are under the double-pulse excitation condition, where the laser intensities for the main excitation pulse (horizontally-polarized, p-pol.) and the pre-pulse (vertically-polarized, s-pol., 4.6 ns in advance of the main pulse) were 0.4 mJ/pulse and 0.1 mJ/pulse, respectively.

One experiment for THz wave emission enhancements was performed under a double-pulse excitation. Figure 4 shows X-ray emission (a) and THz wave emission (b,c) with the pre-pulse (0.1 mJ/pulse, vertically-polarized, s-pol.) with the delay time of 4.6 ns in advance of the main pulse (0.4 mJ/pulse, horizontally-polarized, p-pol.) irradiation. X-ray emission apparently showed an intensity enhancement under the double-pulse excitation, as expected [37,38]. One additional peak at the downstream side was also clearly discernible, as reported recently [43]. With a time delay of 4.6 ns between the pulses, the initial processes of water film ablation and transient surface roughening under action of capillary forces and micro-droplet formation (mist) at the close position to the initial location of the solution surface, all induced by the pre-pulse irradiation, caused a more effective coupling of the main pulse with such a modified solution surface. The enhancement was caused by multiple scattering, local refocusing of light by droplets and the perturbed surface, which resulted in the X-ray intensity enhancement, which can reach an order of magnitude and is useful for practical applications. THz wave emission in the reflection (Figure 4b) was also enhanced about five times, and the profile width under the double-pulse excitation became narrower at 52 μm as compared with the 106 μm width under the single-pulse excitation. The profile of THz wave emission along the *Z*-axis under the double-pulse excitation showed only a single peak at the centre as in the case of the single-pulse excitation, which was different from the profile of X-ray emission with the additional peak at the downstream side. This is consistent with requirement of thermal gradients lasting ∼1 ps for ∼1 THz emission, which are less likely on a fragmented water film, while X-ray emission is maintained by hot plasma and geometrical factors are less important. A detailed investigation is needed using time-resolved shadowgraphy to reveal the geometrical evolution of the disintegrating water surface. In the case of THz wave emission in the transmission, the profile showed an apparent change (Figure 4c), namely the intensity was enhanced more than ten times, and the profile along the Z-axis changed to a single peak from the profiles with a dip at its centre, as shown in Figure 3b,d. Under the pre-pulse irradiation condition, transient surface roughness, droplet (mist) formation and hole formation on the solution flow were expected at a delay time of 4.6 ns [37]. These initial processes of laser ablation induced by the pre-pulse irradiation may cause the enhancements of THz wave emission especially in the transmission direction.

## 4. Conclusions and Outlook

This study reports the demonstration of the dual X-ray and THz wave simultaneous emission from water flow irradiated by focused femtosecond laser pulses in air. Different characteristic features of THz wave emission, which are associated with X-ray emission, in the reflection and the transmission under the single-pulse excitation were clearly revealed. Enhancements of THz wave emission under the double-pulse excitation up to 5–10 times were also shown and indicated that further enhancements of THz wave emission are expected by optimizing the laser excitation conditions. Another option as laser parameters to enhance THz wave emission is laser chirp [24] or double-colour excitation with the fundamental and the second harmonic, expecting efficient augmentation of energy delivery to the target via an optical process such as four-wave-mixing [17]. Various solution samples can be utilised as targets, since X-ray emission from water is also enhanced by the addition of electrolyte [39] and gold nano-particles [40]. Other materials such as bismuth [55] and copper [56] can be also used as targets at the tested high-irradiance conditions. Interaction of the X-ray or THz wave with matter originates with electrons at keV or meV, in other words with electrons bound in inner-shells or with structural absorption resonances, respectively. Based on the basics, X-ray and THz wave science and technology for spectroscopy and imaging have made their progress independently [57,58]. Under experiments in water or at atmospheric pressure, ultrasound emission is also expected [59], and super-resolution photoacoustic imaging is also further developed [60]. With synchronized X-ray and THz wave emission as introduced in this paper, not only for the basic mechanism study on THz wave emission from aqueous solutions based on laser-plasma dynamics, but also combined synchronous usages of X-ray and THz wave or ultrasound are expected to contribute well to studies on nanomaterials from the nano-scale viewpoints to macro-scales.
